# The cognitive science of language diversity: achievements and challenges

**DOI:** 10.1007/s10339-025-01262-z

**Published:** 2025-02-25

**Authors:** Antonio Benítez-Burraco

**Affiliations:** Department of Spanish, Linguistics and Theory of Literature (Linguistics), Faculty of Philology, University of Seville, C/Palos de la Frontera s/n, 41004 Seville, Spain

**Keywords:** Multifunctionality of language, Sociolinguistic diversity, Language typology, Developmental paths to language, Neurodiversity, Non-WEIRD societies/subjects

## Abstract

Linguistics needs to embrace all the way down a key feature of language: its diversity. In this paper, we build on recent experimental findings and theoretical discussions about the neuroscience and the cognitive science of linguistic variation, but also on proposals by theoretical biology, to advance some future directions for a more solid neurocognitive approach to language diversity. We argue that the cognitive foundations and the neuroscience of human language will be better understood if we pursue a unitary explanation of four key dimensions of linguistic variation: the different functions performed by language, the diversity of sociolinguistic phenomena, the typological differences between human languages, and the diverse developmental paths to language. Succeeding in the cognitive and neurobiological examination and explanation of these four dimensions will not only result in a more comprehensive understanding of how our brain processes language, but also of how language evolved and the core properties of human language(s).

## Introduction

Nowadays, diversity has become a major concern for society, and this is also true for scientific research. People are diverse biologically and above all culturally. Social sciences are increasingly acknowledging the value of this diversity, particularly as a primary source for understanding human nature, but also as signal of cultural vitality. This parallels what happens in other fields, most notably biological sciences. Biological diversity is an unvaluable source of data for construing robust hypotheses about the nature of life, the origins of life on Earth, and how life evolves to adapt to different environments. Certainly, biology has made enormous progress during the last decades by focusing on selected animal species, like the fruit fly or the mouse. But the true adaptability of life and the true limits of life cannot be properly understood if we do not also investigate extremophiles, that is, species living in hostile environmental conditions like deep caves, hot springs, or radioactive waste. Likewise, a great deal of our understanding of how evolution works resulted from the examination of domesticated varieties of animals, Darwin’s own work on domesticates being a notable example. But the ultimate mechanisms of evolution would remain quite opaque if the whole diversity of life, past and present, is not considered. The same is (or should be) true for disciplines aimed at understanding our distinctive cognitive phenotype, particularly, language (the hallmark of the human condition), and more specifically, how our brain processes language. Similarly to biological sciences, over the last decades, we have made a lot of progress on clarifying how language is represented in the brain and how this knowledge is computed to allow us to think or communicate. Nonetheless, most of this research have relied on a small set of languages, most notably, widely spoken Indo-European languages, like English or Spanish. Moreover, we have mainly examined the standard language as used by the cultivated speaker in formal settings. By contrast, much less is known about how the brain deals with other typologically diverse languages or even other varieties of the most studied languages, in spite of the wealthy body of research currently available about their structural features and patterns of use. Following with the comparison with biology, it is time for the cognitive science and the neuroscience of language to examine their own extremophiles, domestic varieties, and relict species.

Accordingly, in this position paper, we first highlight the notable extent to which diversity pervades language, from its biological machinery to human societies, and discuss how this pervasive variability has been treated by linguistics to date. We then discuss in some detail four domains of research that are still underexplored by the cognitive science and the neuroscience of language: the diversity of language uses, typologically-infrequent phenomena, non-standard language varieties, and neurodiverse languages. The take-home message will be that although some preliminary work has been done in these areas, we need more research on these issues, particularly, more interdisciplinary research, if we wish to circumvent the limitations and shortcomings of current approaches to the neurobiology of language, and ultimately to gain a more accurate knowledge of how language is processed by our brain.

## The real extent of linguistic diversity

Variation and diversity pervade language. We use language for fulfilling many different functions, from conveying information to others, to structuring our thoughts, to socializing. Humans speak thousands of different languages that are structurally diverse at all levels: phonological, morphological, syntactical, and lexical. In turn, different varieties of each individual language can be found across different social groups, geographical areas, age groups, and so on. Ultimately, differences can be observed from one person to another, and even regarding the same person, as she typically uses her native language(s) differently depending on the conversational setting or the aim of the interactions.

Cognitively, the successful management of all this diversity is challenging. When addressing to others, bilingual people need to select one language or the other depending on the context. But the same happens with speakers of one language when they change from one geographical variety to another, from one sociolect to another, or choose among different registers, depending on if they wish to speak like a father or like a teacher. One can certainly identify a cognitive device (or more properly, a set of interconnected devices) that enables us to acquire and use a language (call it language faculty, human linguisticality, or language-ready brain, as you prefer). This cognitive machinery is supposed to process the core (universal) aspects of a language (phonology, grammar, etc.). Nonetheless, language users typically recruit many other cognitive resources when speaking or signing to others, depending on the kind of language variety they are processing. For instance, in casual conversations, a great deal of the information to be transmitted is conveyed implicitly through implicatures, this making context and background knowledge more important than in formal interactions, particularly when they involve unknown people (Wray and Grace 2007). Not surprisingly then, diverse social and cultural factors act very early as constraints on the interpretation of linguistic meanings (Katz et al. 2004). It is this circumstance that ultimately accounts for the recruitment of extra cognitive resources for creating and interpreting utterances, and accordingly, for the involvement of non-core brain language areas in language processing (Pylkkänen and McElree 2007). Nevertheless, two different persons using the same language variety for fulfilling the same function in the same conversational setting can still process language (slightly) differently. Hence, psycholinguistic responses to the same linguistic stimuli in the same context have proven to be diverse even after carefully controlling for the uniformity of subjects, stimuli, and conditions (e.g. Tanner and Van Hell 2014; Tanner 2019). Likewise, although neurolinguistics has identified regions of interest (RoIs) that activate in response to specific linguistic stimuli, their exact extension, location, and boundaries typically change from one person to another (e.g. Mahowald and Fedorenko 2016). This is suggestive of each person bearing a (slightly) different language faculty, both cognitively and neurobiologically, mostly because of their different developmental trajectories when acquiring their language(s) (Sin Mei Tsui et al. 2021). Overall, the language faculty seems a rather malleable cognitive device that emerges in (slightly) different ways. This type of diversity and variability is even more noticeable in people affected by cognitive conditions impacting on language abilities (e.g., Kjellmer et al. 2012). They bear a different human linguisticality, but still a functional one. This is remarkable if one considers the significant differences that exist between the neurotypical and the neurodiverse brains. Although this will not be part of our main concerns here, even more diversity can be found if one examines other biological components of language “beneath” its cognitive architecture, most notably, genes, which are remarkably pleiotropic (i.e. they contribute to different body functions) and noticeable polymorphic (i.e. some variants can give rise to language/cognitive disturbances, but others result in differences in processing abilities or acquisitional milestones in the neurotypical population) (see Benítez-Burraco 2020; 2023 for review).

Traditionally, all this variation has been regarded a burden, so that researchers have tried to ignore as much of it as possible to reach the fundamental (and purportedly homogeneous and universal) features/properties of language. This tendency can be tracked back to Saussure, who famously focused on languages (*langues*) instead of uses of a language (*parole*) (Saussure 2011), or later to Chomsky, who famously argued (e.g. 1965) that linguistic theory is mostly concerned with ideal speakers-listeners within homogeneous speech communities. Indeed, languages are usually construed as invariant systems emerging from an ocean of regional/social varieties, which in turn result from the levelling of all the idiolectal (i.e. individual) variation. More recently, the same can be said of psycholinguistic and neurolinguistic studies, which mostly aim to determine one common way of processing language for all languages and all human beings. This past approach is understandable and has indeed allowed to gain a lot of progress in our understanding of human languages: their internal structure, their typological patterns, their biological substrate, their developmental paths, and ultimately, the external and internal factors that regulate their use and change. Nonetheless, as increasingly acknowledged by many (if not most) researchers, minimizing the importance of variation in language is also problematic and can eventually result in biased views of what language is and how it is put into use. One reason is that a radical idealization of language phenomena, because of a similarly radical dismissal of their variability, can produce biologically implausible objects/processes, which is fatal flaw for a true neuroscience or cognitive science of language. For instance, Deacon (2005) has put into question that linguistic items and rules, as traditionally described by grammatical theory, reflect the way in which the brain works during language processing. Less radically, Poeppel and Embick (2005: 105) has pointed out that “the fundamental elements of linguistic theory cannot be reduced or matched up with the fundamental biological units identified by neuroscience” (yet, this is not an irresolvable conundrum, as it is possible to spell language in computational terms that can be performed by specific neuronal populations, for instance, via the coupling of brain oscillations, as we discuss below). A second important reason why the cognitive science of language and the neuroscience of language should embrace variation is that the consideration of variation has proven to be helpful for understanding the nature of language at other analytical levels. For instance, the study of language variability within speech communities can help predict and explain language change over time (Weinreich et al. 1968). Since the “brain organization dynamically changes over multiple and spatial scales” (Bassett and Gazzaniga 2011: 201), the consideration of this variability should help understand how language is processed in real time, but also how it grows in the child and eventually, how it developed in the species, as these are just different temporal perspectives of the same neurocognitive phenomenon. Likewise, in biology, intra-individual variation is acknowledged as an essential factor promoting development; in turn, changes in developmental paths account for the evolution of the species (see e.g. Müller 2007). Finally, similarly to language variability, behavioral variability is a precursor (and a predictor) of developmental transitions from one (stable) stage of development to another (van der Maas and Molenaar 1992; Thelen and Smith 1993), with these transitions resulting in speciation events. However, as van Geert and van Dijk (2002: 370) have also pointed out, “developmental theories make little room for variability within individuals as a phenomenon of interest, either as an indicator of development or as cause or condition of change.” This can be indeed said of most current approaches to the neurocognition of language.

This problem becomes exacerbated by the multifactorial nature of linguistic phenomena, which is greater than previously assumed. For successfully dealing with this complex scenario, a truly multidisciplinary approach to the cognitive science and the neuroscience of language is compulsory, both theoretically and methodologically. But this is a formidable challenge. Theoretically, it is necessary to reconcile views of language (linguistic, anthropological, sociological, psychological, physiological, or genetic) that can be very diverse, if not contradictory. Accordingly, in some cases the uniformity of language is a sort of axioma, as with e.g. generativist or paleoanthropological construals of language, which see it as a homogeneous, universal cognitive ability of the species. By contrast, functionalist linguists, anthropologists, or clinical linguists are prone to accept a variable human linguisticality. But reconciling these opposite views could not be enough. It is also urgent to consider theories from other fields, particularly, from biology. For instance, at present it is clear that language emerges from the complex interactions among multiple developmental factors. Accordingly, our view of language will benefit from the consideration of Evo-Devo theories or systems biology approaches in biology, which are aimed at clarifying developmental dynamics (in Sect. "The diversity of language acquisition" below, we characterize both theories in some detail). Methodologically, we need to improve our analytical tools. This certainly entails using facilities and methods from other fields. For example, pragmatics, discourse analyses, and sociolinguistics have developed good methodologies for analyzing how people use different language varieties in different conversational settings. Now we might want to learn how the brain of speakers deal with such diversity, if. e.g. code-switching entails (and results from) a differential brain-to-brain entrainment between them (Pérez et al. 2019). Likewise, we know that each person speaks a slightly different variety of her mother tongue, i.e. an idiolect. Typically, idiolectal variation has been a concern for sociolinguistics, particularly, for sociophonetics, since a great deal of idiolectal features concerns to speech. But we might be now interested in knowing if e.g. idiolectal variation entails (and result from) different neural architectures, brain activities during language processing, or gene expression profiles. Finally, for properly embracing and analyzing this type of variability, we need better tools for processing big data, as in e.g. population genetics studies aimed at uncovering the effect of specific gene variants on language performance in thousands of patients (e.g. Doust et al. 2022 on dyslexia).

Ultimately, this improved approach to the cognitive science and the neuroscience of language can result in a better understanding of causation with regards to linguistic phenomena. Traditionally, language diversity (and language change) has been construed parametrically (e.g. Campbell 1999; Lightfoot 1999). Sociolinguistic variation, which is multifactorial by nature, has been examined through statistical methods mostly. As a consequence, sociological variables cannot be regarded as casual factors of the observed variation; they are just analytical tools (see Milroy and Milroy 1997 for dsicussion). Consider now recent claims that, neurobiologically, language results from the coupling of multiple brain oscillations (e.g. Benítez-Burraco and Murphy 2019; Goswami 2019; Poeppel and Assaneo 2020). Also consider the finding that noise improves the synchronization of the human connectome at different hierarchical timescales (Pang et al. 2021), and more generally, that developmental noise contributes very significantly to the innate variation observed in diverse psychological traits (Mitchell 2022). Neither parametric nor statistical approaches can properly apprehend all this. At the same time, including stochasticity in our neurocognitive models of language demands better analytical tools that can detect and study these effects.

A final concern: past approaches to the neurocognition of language have suffered from a dramatic sampling bias, as they have usually focused on referential functions of language mostly aimed to convey complex propositional contents, the standard variety, the literate speaker, and the neurotypical subject. Even worse, although we have good typological characterizations of the world languages, most of what we know about the cognitive and neurobiological foundations of language results from research examining a small set of languages (usually, English and other widespread Indo-European languages) (see Blasi et al. 2022 for some examples and additional discussion).

Nearly two decades ago, Levinson (2012) regarded this general tendency to dismiss that human cognition is variable and diverse as ‘the original sin of the cognitive sciences’ and urged cognitive scientists to recognize “the true extent of structural diversity in human language [which] opens up exciting new research directions [and] new opportunities for dialogue with biological paradigms concerned with change and diversity” (Evans and Levinson 2009). In this position paper, we agree with this view that a robust neuroscience and cognitive science of language should explore to the fullest the variability that pervades linguistic phenomena. Here we specifically wish to highlight four domains that should be promoted to a more prominent place in the cognitive and neurobiological studies of language. These are the diversity of language functions, the diversity of human languages, the diversity of sociolinguistic phenomena, and the diversity of developmental paths to language, including the neurodiverse trajectories. In what follows, we illustrate the type and degree of variation found within these four domains, suggest possible ways of dealing with the observed variation, with a focus on methodological concerns, and discuss how (and why) properly addressing all this diversity can result in a better comprehension of the nature of human language. By no means we are suggesting that anything has done to date in this line. As we acknowledge below, over the past decade, groundbreaking research on e.g. differential processing strategies in typologically diverse languages has been conducted by Bickel, Sauppe, Stoll, Dediu, Evans, Christiansen, Everett, Levinson, or Nuñez, to name a few. Likewise, the impact of sociopolitical factors on our linguistic cognition has been addressed by Rubio-Fernández or Trudgill, among others. Therefore, what we mostly aim in this position paper is to highlight the aspects of such a neuroscience and cognitive science of linguistic variation that still need to be improved (methodologically and theoretically), and to suggest that an unitary explanation of all these apparently disparate phenomena can be achieved if we focus on some basic processing strategies, like memory types.

## The diversity of language functions

Traditionally, a main concern for the cognitive science of language has been how words are stored in the brain and how we combine them to produce complex sentences aimed to convey equally complex propositional contents. However, language is mostly employed for socializing, to the extent that recent quantitative analyses suggest that up to 85% of casual conversations gravitate around social matters (Szala et al. 2024). The type of language we use for fulfilling these other functions can differ significantly from the kind of language aimed at transmitting propositional information. Accordingly, it usually features less syntactic complexity, reduced semantic compositionality, with a greater reliance on chunks, formulae, and idioms, and increased contextual dependence (Van Lancker Sidtis 2004; Wray and Grace 2007). These differences can be certainly expected to impose different cognitive demands, although some overlap with the processing of referential, propositionally complex language certainly exists too. For instance, cognitively, chunks and formulaic language are less costly in terms of working memory demands (Hartmann 2013; Rabinovich et al. 2014). This is explained by their less compositional nature (more complex structures demand more working memory resources), but also because chunks facilitate predictions about the grammatical nature of next items in the discourse (see, e.g. Coopmans et al. 2022, for sentence-final verbs). At the same time, because of their limited compositionality, their processing involves a reduced participation of the procedural memory, which is more important for rule learning and use, but a greater contribution of the declarative memory (Ullman 2004). Likewise, one could expect a reduced involvement of executive functions in the processing of non-referential uses of language, which are instead more important for formulating propositional contents (Barker et al. 2020). Also, because of their more affective nature, non-referential uses of language can be expected to engage cognitive mechanisms that are more directly related to perception, emotion, or sensorimotor issues (Wilson and Carston 2019). There is indeed evidence of a person’s emotional state impacting, specifically, on semantic and syntactic processing, with this effect being context-dependent, particularly, in the case of semantic processing (Chwilla 2022). A similar impact could be thus expected at the cognitive level. Overall, we can safely assume that language processing by the brain will change according to what we use language for, with the core components of our language faculty interacting with different non-linguistic systems depending on the uses given to language.

As expected, the differences that are observed at the cognitive level can be tracked to the neurobiological level. Referential uses of (complex, compositional) language recruit brain areas around the classical language network, which is mostly left-lateralized (see Jung-Beeman 2005; Skeide and Friederici 2016; Friederici et al. 2017; Van Lancker Sidtis and Sidtis 2018; Barker et al. 2019 for details). This language network also supports referential uses of sign languages (Trettenbrein et al. 2020). The neurobiological picture is significantly different when one considers other types of language. Accordingly, less compositional linguistic items, like idioms, which are more frequent in informal, emotionally-charged uses of language, usually result in a bilateral activation of the language areas (Hertrich et al. 2020). Sidtis has characterized this (partial) neuroanatomical dissociation between compositional and formulaic language in terms of a ‘dual process model of language’. According to his view, compositional language relies more on the left hemisphere, while idiomatic speech is supported by right areas to a greater extent (see Sidtis et al. 2018; Van Lancker Sidtis and Sidtis 2018, for details). Likewise, more hierarchical syntactic structures (of the type: *The crisis was solved*), which are typically found in formal language with referential uses, engage more left-lateralized brain areas, compared to less hierarchical structures (of the type: *Problem solved*), which are more typical of informal language with emotional uses. These structures result in a reduced activation of some classical language areas in the left hemisphere, including Broca’s region (Progovac et al. 2018). Overall, there is a greater involvement of the right hemisphere in the processing of figurative language, implicit meanings, background knowledge, discourse contexts, and pragmatic interpretations, which are all aspects that are more relevant for non-referential uses of language (Ferstl et al. 2008).

Ultimately, if we wish to discover the principles that govern language processing by the brain, we need to pursue a truly contextualized cognitive neuroscience of language. Even in the case of research that focuses on informal, non-referential uses of language, most studies have been conducted in decontextualized settings (e.g. laboratories). Nonetheless, increasing psycholinguistic and neurolinguistic evidence suggests that the context, broadly construed, interacts notably with how stimuli are processed, this resulting in context-dependent changes in the activation patterns of language regions, and the recruitment of additional brain areas (see Willems and Peelen 2021 for a more detailed discussion).

A final point: it might well be the case that the sort of language items and structures that are commonly found in non-referential uses of language are more tractable by neuroscience than the items and structures that are usually found in referential language aimed at conveying complex propositional meanings. The reason is that the latter is more seriously hindered by the widespread problems of granularity (i.e. the circumstance that whereas language theory examines very detailed language phenomena, neuroscience studies broader language distinctions) and incommensurability (i.e. the circumstance that the units and computations characterizing language cannot be reduced or matched to the units and processes of neuroscience) (see Poeppel 2005; 2012 for discussion). By contrast, reduced syntactic structures that, as noted, are commonly found in non-referential language, depend on more specific, less distributed neuronal components. Examples of these structures are small clauses of the type: *Book found* or *Work done*, which contrast to their more hierarchical counterparts: *The book was found*; *The work was done* (also of interest are verb-noun compounds like *kill-joy*, which contrast with their more hierarchical counterparts: *joy-kill-er*). Because these reduced syntactic structures have been claimed to be evolutionarily older (Progovac 2015), we expect them (as well as non-propositional language, more generally) to show a greater continuity with other species and to be more tractable in animals (including the generation of reliable animal models of human language). As recently claimed by Benítez-Burraco and Progovac (2024), examining emotional structures/uses of language should not only help us improve our understanding of how human cognition works, but achieve more robust linking theories between theoretical neuroscience and neuroscientific methods.

## The diversity of human languages

Humans speak around 7.000 languages (and sign nearly 200). Linguistic typology has contributed very significantly to identify the core structural properties of human languages, the aspects in which languages tend to diverge, and the phenomena that can be regarded as infrequent, or absent in the world languages. A hundred years ago, our understanding of language was fatally biased by our limited knowledge of this diversity, as research had mostly focused on a bunch of privileged (Indo-European, principally) languages. Today, we have rich databases of typological information (like WALS or Grambank), but also equally rich databases of the sociopolitical and cultural diversity of human groups. This has notably contributed to improve our understanding of the diversity of human languages, but also of the factors external to language that trigger, limit, or modulate this diversity. Nonetheless, the cognitive science of language still suffers from similar shortcomings and limitations as the XIX-century typology, since our view of how the brain processes language mainly builds on findings from research conducted in a limited number of world-wide languages, mostly WEIRD (i.e. Western Educated, Industrialized Rich Democratic) languages, as recently noted by Blasi et al. (2022). An additional concern, still to be clarified, is to which extent, if any, our biological diversity contributes to this type of diversity.

Gradually, some minority languages are being examined by the neuroscience of language. The potential benefits of this change of paradigm are illustrated by studies like the one recently conducted by Malik-Moraleda et al. (2022), who described the brain substrate of 45 languages from 12 different language families. This research uncovered a common functional language network for typologically-diverse languages, and supports the view that all languages might share a common structural skeleton which is processed by the same core brain regions. However, likewise linguistic typology, the neuroscience of language would also benefit from conducting fine-grained analysis of selected language-specific phenomena, particularly, exceptional phenomena, because, as stressed by Perkins (1988), exceptions (or rarities) are the elements that test our theories best. In the domain of linguistic typology, Cysouw and Wohlgemuth have noted that the features and properties found in very few languages (usually referred to as *rara* or *rarissima*), “can tell us as much about the capacities and limits of human language(s) as do universals” (2010:1). Likewise, in his insightful *Syntactic Nuts* (1999), Culicover pointed out that language learning mechanisms are capable of accommodating not only universal properties, but also language irregularities, exceptional and marked cases, and idiosyncratic features. Accordingly, in parallel to a refinement of our understanding of the core language network, exceptional phenomena of the sort mentioned by Culicover should be a necessary focus of attention for cognitive sciences too. In other words, we need more research aimed at knowing how our brain deals with the variable cognitive loads and demands imposed by different types of linguistic phenomena of typological interest, including those like *rara* that are less frequent and seemingly, less functionally motivated, in particular, less optimized in terms of learnability and informativeness (see e.g. Carr et al. 2020 for semantic categories, or Saldana et al. (2019) for hierarchical structures). Even more, the neuroscience of language should analyze as well what Adli et al. (2015) have called *language-specific rara*, that is, infrequent language phenomena within particular languages. Such language-specific rara are usually maintained via social pressure and sociolinguistic variation, thus imposing additional cognitive loads on speakers, as we will discuss in the next section.

Just to give a flavor of this new neuroscience of language diversity, and how typologically-diverse language phenomena can impact to different degrees on different cognitive domains (from perception to memory to social cognition), let us first consider the case of word order. Speakers of languages with different basic word order (e.g. S(ubject) O(bject) V(erb) vs. SVO) have been shown to exhibit a differential ability at recalling initial vs. final stimuli (Amici et al. 2019). Likewise, speakers of languages with less harmonic structures (e.g. with a Adj(ective) N(oun) order in the noun phrase, but with a VO order in the verbal phrase) tend to show a less marked regularization bias (i.e. the tendency to regularity when dealing with structural rules) (Culbertson 2012). Also, different languages can impose different patterns of conceptualization and categorical representation on the knowledge about the world facts, mostly because of cultural constrains, this in turn affecting differently the automatization and the acuity of perceptive abilities (Kemmerer 2006). Ultimately, language-specific structural constraints can be associated with a differential involvement of specific cognitive functions in language processing, with language features that are more costly to process and learn resulting in the creation of “cognitive gadgets” through modifications in learning and data-acquisition mechanisms (Clarke and Heyes 2017). A second example concerns to typological rara like ideophones. Ideophones are conventionalized, structurally (usually, phonetically) marked words associated with vivid sensory images, which are typically employed to depict qualities of things or events, and which are a grammatical category in only a small set of languages (see Dingemanse 2012 for a review). The similarities found by typological research among the ideophones of the world languages is seemingly explained by their closer relationship to the sensory and motor systems within human cognition, which are rather invariant (De Varda and Strapparava 2022). Yet, some within- and across-language variability in how ideophones are built and used has been documented too (Dingemanse 2012). This is certainly of interest for a cognitive science of language. Neurolinguistic research on ideophones is growing slowly. Preliminary findings suggest that ideophones activate different brain areas to non-ideophonic language (e.g. Lockwood and Tuomainen 2015). Specifically, they activate sensory or motor areas related to their meanings. Accordingly, in a recent study, Dewey et al. (2024) observed that ideophones conveying visual, motion, or sound meanings activate areas of the brain that are associated with visualization, motion, and sound processing and production, respectively, even if presented isolated to participants. This pattern of activation has been characterized as (quasi-)synesthetic, i.e. cross-modal (Osaka et al. 2004; Osaka 2009). Overall, knowing more about how the brain processes ideophones would be a valuable window into the mechanisms that link language forms to their conceptual representations, and particularly, to the emotional and affective values of words (Perniss et al. 2010).

A final remark: most typologists think that present-day language diversity is the outcome of how languages changed in the past; that language change depends, in turn, on how languages are acquired and used; and that language acquisition and use are ultimately subject to functional constrains, most of which are cognitive by nature, including general cognitive biases that favor e.g. systematicity, salience, or harmony in language structure (Culbertson 2012; Culbertson et al. 2013). Accordingly, if we increase the number and the diversity of languages (and linguistic phenomena) under the scrutiny of a neuroscience of language, we will achieve a better understanding not only of how the human brain processes language (diversity), but also of the cognitive biases that systematically guide language change, which, as noted, are the ultimate source language universals. In other words, other disciplines, particularly, linguistic typology can be expected to benefit from this new approach to language diversity.

## The diversity of language varieties

As noted enough in Sect. "The real extent of linguistic diversity", languages are idealized linguistic varieties that result from levelling an ocean of underlying linguistic diversity. In particular, it is well-known that the distinction between languages and dialects mostly depends on extralinguistic factors: political, economic, cultural, or historical (e.g. Serbian and Croatian are now regarded as different languages, despite their mutual intelligibility; by contrast, Andalusian and Castilian are named as two different dialects of Spanish, even if they are partially unintelligible). This means that our understanding of the cognitive underpinnings of language will equally benefit from the study of the varieties of each particular language. Traditionally, intralinguistic diversity has been a concern for sociolinguistics and other close disciplines, most notably dialectology and discourse analysis. Nonetheless, these disciplines have mostly focused on the factors external to language that impact on the structural features and patterns of use of a language (like gender, social class, age, or conversational settings), with the aim of seeking for correlations (and ideally, causation) between the two domains. Yet, as we noted for typological diversity, this intralinguistic variability is expected to result in variable cognitive demands. As pointed out by Thomas (2011:703), “language does not operate without constant cognitive interface with sociolinguistic knowledge”. This ultimately means that when producing or understanding language, we also need to compute a myriad of linguistic variants resulting from the dozens of sociological and contextual factors that impact on interactions through language. These include registers, codes, styles, or sociolects. Nevertheless, sociolinguistics has largely ignored this cognitive side (although see Kristiansen 2008, or Moreno-Fernández 2016, for some groundbreaking work), pretty much as cognitive sciences have largely ignored this type of linguistic diversity. Certainly, in the domain of pragmatics, some of these effects have been studied in some detail, but cognitive pragmatics is mostly concerned with the mental processes involved in intentional communication, like the access to the implicit meanings conveyed by utterances, but not properly with the sort of macrovariation resulting from sociological factors (Bara 2011; Schmid 2012). Accordingly, intralinguistic variation should be promoted to a more prominent place in ongoing research about the neuroscience and the cognitive science of language.

By way of illustration, consider social indexicality. This type of indexicality refers to the conventionalized link between language features and social dimensions or constructs, like gender, social class, or ethnicity (Campbell-Kibler 2021). Social indexicality has been shown to impact on the cognitive processing of language in many diverse ways. One way is through predictive actions, both during language production and language processing (see Pickering and Garrod 2013, for details). In brief, the more social salience a language feature has, the greater cognitive availability it shows (D’Onofrio 2021). In turn, this effect can be related to memory issues, as some social indexes are stored in the long-term memory (like the phonetic cues characterizing sociolects or speech styles), whereas others are stored in the short-term memory (like some of the phonetic cues involved in the negotiation of conversational turns, as the pitch updrift at the end of an utterance when we wish to hold the floor) (see Foulkes 2010 for details). Additionally, our social expectations can bias our memory , with social prominence usually resulting in more efficient storage, improved processing, and increased intelligibility (Foulkes 2010; Thomas 2011; Levon and Fox 2014). Since most if not all language features index some type of social information (see Foulkes and Docherty 2006, for discussion), this sort of effects can be expected to have some measurable impact on our cognition, and accordingly, to be of interest for the neuroscience of language. Ultimately, sociologically-motivated preferences for some language features result in their conventionalization (Sinnemäki 2014). In other words, together with factors related to language use like learnability or informativeness, sociological factors are a principal source of linguistic traits of typological relevance, mostly through their impact on language change.

The type of research sketched above can (and should) be extended to other types of intralinguistic variation, specifically to dialectal variation, which is known to also impact on dimenssions like cognitive flexibility or accessibility during language processing (Levon and Buchstaller 2015). Nonetheless, as recently pointed out by Majid (2023), most research on the neuroscience of language has been conducted with participants who are literate, received extensive formal education, are endowed with notable technological abilities, and belong to privileged social groups. This means that we know less about language processing by speakers of non-standard varieties of a language... and this is true even for WEIRD-languages. Fortunately, things are changing, even if slowly. For instance, Byrd et al. (2023) have found that some of the dialectal differences between African American Vernacular English (AAVE) and Mainstream American English (MAE) have a measurable impact on sentence comprehension by children. Likewise, according to the review by Lønes et al. (2023), dialectal words are differentially represented and selected for production in the brain compared to the standard lexicon.

## The diversity of language acquisition

As noted in Sect. "The real extent of linguistic diversity", a great amount of the variability in the neurocognitive foundations of language pertains to differences in how language is acquired. People are biologically diverse and they are exposed to variable linguistic inputs (including the sort of variation discussed in the sections above). At the same time, changes in language acquisition patterns promote language change, which in turn accounts for present-day language diversity, which are the main concern for linguistic typology. This means thatexamining the variability in language processing during development is not only important for gaining a better view of how our brain processes language more generally, but also for understanding the causes of language diversity. Nevertheless, research on the neuroscience of language has typically focused on been neurotypical adult speakers. Yet, the fields of language acquisition and clinical linguistics are lively fields, and neurobiological and cognitive perspectives are increasingly common nowadays. Research has uncovered a notable diversity in language acquisition patterns and trajectories by both children and neurodiverse people. This suggests that the cognitive and neurobiological diversity of children and neurodiverse individuals is going to be greater than the variability observed in adult neurotypical subjects. This type of variability should also be a central concern for a neuroscience of language. Not surprisingly, studying this variability poses notable challenges, both methodologically and theoretically. In this section, we briefly comment on the main findings in this domain, as well as on such difficulties.

Over the years, we have certainly gained a good understanding of how children acquire their mother language(s). We have learned that the process can differ significantly from one child to another, in terms of pace and developmental trajectories, and ultimately, the cognitive strategies used for mastering the language(s) (see Bates et al. 1988; Fenson et al. 1994; or Slobin 1987 for classical accounts). For instance, comprehension abilities can outperform production abilities to different degrees, as in *early talkers* vs. *late talkers* (Fenson et al. 1994). Likewise, children can follow different strategies to generate their first utterances. Hence, whereas some children produce well-articulated utterances from the scratch, others rely more on phonological templates, with these ‘prosodic bootstrapping’ strategies typically resulting in more variability (Vihman 1996). Later, when longer utterances are produced, some children rely on purely combinatorial mechanisms, whereas others adopt a ‘slot and frame’ strategy (Bloom et al. 1975; Veneziano and Sinclair 2000). The structure and the nature of the early child lexicon is also rather variable, with some children preferring individual words to chunks, and vice versa, as in *noun-lovers* vs. *noun-leavers *(Lieven et al. 1992). A notable diversity is also observed during the last stages of language acquisition, affecting to different aspects of language structure (e.g. overregularization patterns or word order preferences) and language use (e.g. the acquisition of pragmatic competence) (Ninio and Snow 1996). Even the individual path to language is rather variable (Chabon et al. 1982; Ruhland and van Geert 1998; Fenson et al. 2000; van Geert and van Dijk 2002).

As noted at the beginning of this section, this variability in language acquisition can be explained by the variability in the Primary Linguistic Data (henceforth, PLD) to which children are exposed during growth, and by differences in their cognitive abilities. The nature of the PLD, particularly, the language used by caregivers has proven to have an impact on the acquisition of many components of language, as well as the strategies followed by children for producing their own utterances (Brice Heath 1983; Galloway and Richards 1994; Hart and Risley 1995; Pine 1995; Huttenlocher et al. 2002; Huttenlocher et al. 2010). The amount and properties of the PLD typically depend on extralinguistic factors, such as the socioeconomic profile of the caregivers (Huttenlocher et al. 2002; Hoff 2003; Huttenlocher et al. 2010), or the presence of other siblings or peers (Kerswill 1996; Hoff-Ginsberg 1998). With regards to the cognitive differences between children, their impact on language acquisition has been widely recognized (Plomin and Dale 2000; Stromswold 2001; Colledge et al. 2002; Plomin and Kovas 2005). Neurobiologically, language acquisition entails profound changes in the child brain, both structural and functional, which are still poorly understood. As a rule, the same brain areas are involved in language processing across all developmental stages, although some differences with adults can be found too (Dehaene-Lambertz et al. 2002; 2006; Kuhl 2010). Interindividual variability is also notable at this neurobiological level (Kidd et al. 2020).

As pointed out in Sect. "The real extent of linguistic diversity", this significant developmental diversity is even more noticeable in people affected by cognitive conditions impacting on language abilities. Our cognition and our brain tend to react adaptively to ontogenetic damage, either acquired or resulting from genetic disturbances. Additionally, people with these conditions are usually exposed to different PLD compared to the neurotypical population, with this input being adapted to their (dis)abilities (see e.g. Rowe et al. 2009 on acquired language disorders in children). Both factors result in increased variability, of true interest for a neuroscience of language. For instance, in acquired language disorders, early and extended brain lessons can produce a notable reorganization of the languages areas, including the transfer of language functions to the right hemisphere (Vargha-Khadem et al. 1985; Duchowny et al. 1996; Staudt et al. 2002; Lidzba et al. 2006). Even narrow and/or late injuries typically result in peri-lesional reorganizations and a compensatory activation of larger networks (Müller et al. 1999). As a consequence, children affected with brain injury show more variable language acquisition patterns than their neurotypical peers (Feldman 2005; Rowe et al. 2009), with the etiology and the timing of the lesion(s) being two major predictors of their language outcome (Loonen and van Dongen 1990; Chilosi et al. 2008). This variability is even more noticeable in the case of developmental language disorders, which result from genetic or epigenetic anomalies. In these conditions, language acquisition is usually delayed, but it can be deviant too. Delays, asynchronies, and/or deviances are also observed at the cognitive and brain levels throughout development. In fact, significantly preserved language abilities frequently mask notable cognitive differences, if e.g. compensatory strategies are used for processing language. In turn, these cognitive differences can result from even greater structural and functional differences in the brain. Additionally, the same disorder can present differently at different developmental stages. As noted by Annette Karmiloff-Smith, who conducted extensive research on one of these conditions, namely, Williams Syndrome, (WS), “to understand developmental outcomes, it is vital to identify full developmental trajectories, to assess how progressive change occurs from infancy onwards, and how parts of the developing system may interact with other parts differently at different times across ontogenesis” (Karmiloff-Smith 2009: 58). And this needs to be done at different levels: neurobiological, cognitive, and behavioral, since there is typically an indirect translation between the three of them, so that e.g. nearly normal behavioral scores can camouflage very different cognitive and neuronal processes (see Donnai and Karmiloff-Smith 2000, or Karmiloff-Smith 2008 apropos WS).

The kind of variation we have highlighted in this section probes to be elusive and difficult to analyze. Some methodological and theoretical considerations can help deal with it. Methodologically, we need more precise neuroimaging facilities, more accurate tools for recording speech, and more generally, better ways of registering and studying language processing in real time. We also need more research on language acquisition by children from non-WEIRD societies (see Bunce et al. 2020; Bergelson et al. 2022 for promising examples), but also more studies about how language is acquired by children not belonging to privileged groups within Western societies. We likewise welcome research aimed at clarifying language acquisition and language processing by people with cognitive conditions impacting on language, particularly if speaking minority languages. As recently noted by García et al. (2023), there is a shortage of linguistic diversity in research clinical linguistics, despite the same disorder can present with different language problems depending on the language spoken by the affected people.

Nonetheless, we also need better theoretical approaches to the neuroscience of variation, particularly, if we wish to capture (and explain) the diversity of developmental paths to language, either neurotypical or neurodiverse. Increasing evidence suggests that the child linguisticality is not just a thumbnail of the adult linguisticality, just like language disorders cannot be merely construed as breakdowns of the neurotypical faculty of language. Moreover, as noted enough, language development and language processing involve the interplay of a myriad of assorted factors, internal and external, and this certainly contributes to (and explains) the variability at every level, from genes to speech communities. Thinking of a far-reaching neuroscience of language, we need, specifically, theoretical models of language that can account for the diversity observed from genes to language output, throughout all developmental stages, and in both neurotypical and neurodiverse groups. For instance, one could adopt a systems biology approach to the neuroscience of language, aimed at studying the whole dynamics of the biological components of language, with a focus on emergent properties (Kitano 2002). Or one could adopt an evo-devo approach to the neuroscience of language. Evo-devo (from evolutionary developmental) theories in biology argue for a deep link between evolution (the ‘evo’ side) and development (the ‘devo’ side), with evolutionary innovations resulting from changes in the developmental instructions needed for constructing an organism (Hall and Olson 2006; see Griffiths 2007 for an evo-devo neuroscience). The benefits of adopting such an evo-devo perspective are apparent if one recalls, in particular, the main findings of clinical linguistics in the domain of developmental language disorders (discussed in more detail in e. g. Benítez-Burraco 2018; 2023). First, the neurodiverse brain is not just an admixture of impaired and preserved components, but a brain which is organized differently, with gene mutations (and other developmental perturbations) exerting diffuse effects on the whole system, and accordingly, impacting even if subtly, on most if not all cognitive capacities/abilities. Accordingly, developmental language disorders are better characterized in terms of abnormal associations across diverse cognitive domains than in terms of dissociations between specific domains. Second, in developmental disorders, language deficits typically result from the impairment of broader cognitive functions. Third, despite this circumstance, each disorder usually exhibits a disorder-specific linguistic profile (even if, as noted, its symptoms are usually heterogeneous, shared with other disorders, and changeable throughout development). Fourth, simultaneously, some language deficits are rarely observed in developmental disorders, while other aspects of language are impaired in most if not all conditions; putting this differently, some components of language seem to be particularly vulnerable to damage, whereas others are rather preserved even despite notable developmental perturbations. At the same time, the number of disorders is far smaller than the number of etiological factors involved. It is this complex scenario that can be apprehended by the “devo side” of evo-devo theories. Accordingly, the small number of disorders attested by clinical linguistics are the only possible phenotypes within the adaptive landscape of human language (following Arnold et al. 2001; or Erwin 2017). Familiar concepts in evo-devo theories, like *canalization* (i.e. the generation of one consistent, or robust phenotype regardless of the existing genetic and environmental variation) or *developmental plasticity* (i.e. the generation of phenotypes from the same genotype in response to environmental changes during development) can nicely describe and explain most of the findings by clinical linguistics (and by research on child language more generally). Hence, the neurotypical linguisticality could be regarded as the result of the successful canalization of the otherwise widespread developmental noise (e.g. gene mutations or minor brain anomalies). By contrast, whereas developmental language disorders could be construed as suboptimal canalizations of more severe developmental disturbances (e.g. deleterious gene mutations or generalized brain damage).

With regards to the "evo side" of such an evo-devo neuroscience of language, one could argue that the aspects of language that are impaired in most, if not all, disorders are the components of our linguisticality that evolved later. The reasons is that recently evolved neuronal devices are typically endowed with weaker damage protection mechanisms, hence their reduced resilience to ontogenetic perturbations (see Toro et al. 2010 for autism; or Pattabiraman et al. 2020 for a general discussion). More generally, genetic changes resulting in evolutionary advantages (like language) might persist even if they also contribute to diseases (like autism or schizophrenia) (see Sikela and Searles Quick 2018 for discussion). Overall, one can confidently expect that the genes that were involved in the recent evolution of the human brain, and human linguisticality more specifically, are also key etiological factors of language disorders, and cognitive conditions impacting on language more generally. For the sake of illustration, let us consider complex syntax. Aspects of complex syntax like embedding are particularly problematic for most neurodiverse people. Cognitively, they involve many assorted representations and computations. Neurobiologically, they depend on intricate interactions between diverse brain regions, with some connections being evolutionarily recent, as the connectivity of the Broca–basal ganglia network, which has been bolstered in the line of descent of humans (and Neanderthals) (see Benítez-Burraco and Progovac 2023 for a detailed discussion).

Ultimately, one could advocate for an eco-evo-devo-approach to the neuroscience of language. The “eco side" means that the environment (broadly construed, this including the physical, but particularly the social environment) should be promoted to a more prominent place in our neurocognitive explanations of how language emerges, both developmentally and evolutionarily. Systems biology, which construes organisms as open systems in constant contact with their environment, has the methodological and conceptual tools for properly capturing the impact of e.g. the ariable PLD on the developing brain. Likewise, eco-evo-devo theories ould properly account for the effects of environmental modifications on brain development, with these developmental changes fostering in turn the evolution of the brain towards a language-ready brain. A reason is that according to eco-evo-devo theories, organisms evolve as a result of the interactions between their genes, their developmental paths, and the environments in which they live (Abouheif et al. 2014; Gilbert et al. 2015). Along these lines, Benítez-Burraco and Murphy have recently argued for a systems biology/eco-evo-devo approach to the neuroscience of language that focuses on brain oscillations, which are primitive components of brain function with a notable evolutionary continuity. According to these authors, brain oscillations could be the best biological component of language for defining the adaptive landscape of language growth in the species, either neurotypical or neurodiverse (for details, see Benítez-Burraco and Murphy 2019; or Benítez-Burraco 2020).

To finish, we wish to highlight that this broader view of the neuroscience of language would be in line with modern views of the relationships between human cognition, language diversity, and our cultural niche. For many years, the mainstream view in language sciences has been that the core properties of human languages are mostly insensitive to our physical and social environment, as they are imposed by our brain anatomy and physiology. Increasing evidence suggests, however, instead that language features can also impact on our mental architecture, as we discussed in detail in Sect. "The diversity of human languages". Additionally, some components of our physical and social environment shape sometypological features of languages. Familiar examples are the negative effect of cold and dry climates on the phonological use of pitch (Everett et al. 2015) or the negative effect of the number of speakers on morphological complexity (Lupyan and Dale 2010). These effects are also of interest for a neuroscience of language, since explanations of this adaptive response of languages to their social niche have a cognitive dimension, including differential language learning abilities (of children vs. adults), differential processing abilities, or a different reliance on memory types, as we elaborate in more detail in the next section.

In summary, the times are finally propitious for a neuroscience of language that construes the different modes of language processing as the result of the interplay between the (constrained) variability of our cognitive architecture and the permanent changes in our physical and social environment.

##  A unified neuroscience of linguistic variation?

It should be now clear that the cognitive science of language cannot regard variation as an artefact or even as a burden, but as an intrinsic property of language. As discussed, this notable variability in language facts is a consequence of the multifactorial nature of the cognitive phenomena related to language, but also from the non-linear, stochastic relationships that exist between the myriad of internal and external factors that are involved in language development, processing, and evolution. We also concluded that this variability is perhaps one order of magnitude larger than previously assumed, and that for properly capturing and explaining it, we might need improved methodological and theoretical approaches phenomena like stochasticity, emergency, canalization or developmental plasticity. A final lesson of our previous discussion is that we can expect that all this diversity (from the multiplicity of languages, to the multiplicity of varieties and uses of a particular language, to the multiplicity of developmental paths to language) maps onto (and is explained by) a diversity of cognitive states and processes. But we should equally expect that this mapping converges on specific cognitive mechanisms, this ultimately reinforcing the unitary nature of language phenomena.

One instance of such convergences might concern the types of memory involved in language processing. In a series of papers, Ullman (e.g. 2004, 2016) has argued that two different memory systems, namely procedural and declarative, support different dimensions of language. Procedural memory is involved in the compositional, rule-governed aspects of language, whereas declarative memory stores word meanings, but also idiosyncratic or even opaque linguistic phenomena, like irregular morphology, idioms or “language chunks” (see also Lum et al. 2012). Our declarative memory is also notably sensitive to the context, hence it is important for non-referential uses of language (Duff and Brown-Schmidt 2012), and for figurative language (see Ullman 2015 for details). This neurocognitive model of language has been also used for explaining other dimensions of language diversity, specifically, the trajectories followed by first and second language learner (Ullman 2015; Hamrick et al. 2018), or the nature of language disorders (Ullman et al. 2020; Earle and Ullman 2021). Consider language acquisition. Our lexical knowledge is stored in our declarative memory only, both during first and second language acquisition. By contrast, grammar abilities can be stored in these two memory types. Specifically, in the case of second language learners, grammar initially depends on the declarative memory only, but the procedural memory gets more involved as the competence in the second language increases. As noted by Earle and Ullman (2021), these findings are consistent across different languages, language families, linguistic structures, and language tasks. With regards to language disorders, they can be construed as the outcome of different types of damage in the procedural circuits, with this circumstance accounting for their commonalities and comorbidities.

Recently, Chen et al. (2023) have suggested that Ullman's model could help understand also aspects of the sociolinguistic typology of the world languages. Specifically, they have argued that declarative memory could be potentiated in native speakers of the languages spoken by close-knit (or esoteric) societies. The reason is that these languages rely more on formulaic/memorized language chunks and are richer in idiosyncratic and irregular phenomena. By contrast, native speakers of the languages spoken by open (or exoteric) societies are expected to exhibit increased procedural memory abilities, since these languages exhibit a more complex and regular morphosyntax, as well as increased compositionality. Chen et al. (2024) have called these two language types Type S languages and Type X languages, respectively. As discussed by several authors (e.g. Bolender 2007; Wray and Grace 2007; Trudgill 2011), the reasons why these two classes of languages exhibit these distinctive, partially opposite features are mostly extralinguistic by nature. Hence, the greater syntactic complexity of Type X languages is seemingly due to the fact that they are frequently used for conveying complex propositional contents to strangers. By contrast, the abundance of idiosyncratic speech in Type S languages is seemingly accounted for their frequent usage as identity markers. Now, if a society evolves from esoteric to exoteric, its language is expected to change to a Type X language, which demands more procedural abilities. Conversely, if a society evolves increasingly esoteric, speakers will need to rely more on their declarative memory abilities for processing their language, since this will be changing to a Type S language. One could even imagine that societal changes that impact on these two types of memories contribute, even if subtly, to this transition from one typological type of language to the other. Some activities certainly potentiate our procedural memory abilities (e.g. working on an assembly line), whereas others increase our declarative memory (e.g. working as a clerk, since you need to remember dozens of names and figures). Accordingly, speakers with increased procedural memory abilities will find Type X languages more learnable and tend to potentiate their distinctive features, whereas people with stronger declarative memory abilities will favor Type-S language features. This neurocognitive model could be also applied to the examination of the varieties of one single language, since all languages are expected to exhibit both esoteric varieties (used in familiar settings) and exoteric varieties (used in formal settings or when speaking to unfamiliar people), with the former relying more on declarative mechanisms, and with the latter favoring procedural mechanisms.

Finally, supporting this convergence of diverse types of language diversity on unique cognitive mechanisms (specifically, on these two types of memory), procedural and declarative mechanisms exhibit a strong continuity with animal cognition (stronger indeed than language as such). At the same time, procedural circuits have evolved significantly in our lineage, this potentially accounting for our species-specific enhanced syntactic abilities. As mentioned in Sect. "The diversity of language functions", the connectivity of the Broca’s area (and other cortical regions) with the basal ganglia (and other subcortical areas) has been bolstered relatively recently during hominin evolution (see Lieberman, 2000, 2015; Ullman 2006; Enard et al. 2009; Hillert 2014 for details). This neurobiological change could be a signature of the potentiation of the procedural mechanisms that ultimately enabled more complex forms of syntax, including those relevant for the efficient transmission of propositional contents. Procedural memory is mostly located in the basal ganglia and associated circuitry (see Ullman et al. 2020 for details). But the complexity of the computed sequences depends on our working memory, which is located in different cortical areas. According to Balari and González (2013) and Balari et al. (2013), evolutionarily, the sequences generated in the basal ganglia ould be manipulated beyond a strictly lineal computational regime (i.e. hierarchical constructions appeared) only when our brain attached more working memory resources to the core (i.e. subcortical) procedural mechanisms. These authors have hypothesized that this happened when the cortex grew larger in the human lineage, so that cortical and subcortical areas became more interconnected via connectional invasion. Nonetheless, Benítez-Burraco and Progovac (2021, 2023) have linked this neurobiological change to our evolutionary trend towards a less aggressive phenotype and a more prosocial behavior, in the line of the self-domestication hypothesis of human evolution (Hare 2017). In brief, the reduction in reactive aggression in response to environmental triggers would have resulted in an enhanced cortical (prefrontal) control of subcortical (striatal) areas, being the potentiation of our syntactic abilities a side effect of this process.

## Conclusions

In this paper, we have urged cognitive science to reinforce its commitment to study language variation in depth, from genes to human societies. Specifically, we have commented on the benefits for the neuroscience of language of examining i) other language uses besides the transmission of propositional contents, ii) typologically-diverse minority languages and typological rara, iii) varieties of well-studied languages (including vernacular varieties and contextual varieties), and iv) the diverse developmental paths to language, by neurotypical WEIRD subjects, but also by non-WEIRD neurotypical individuals and by neurodiverse people speaking WEIRD and non-WEIRD languages. This approach is expected to improve our current understanding of the cognitive principles that govern language. One reason is that this type of diversity encompasses settings, structures, and uses that are closer to the experience that most people have of language in their daily life (e.g. relaxed conversations between close friends sharing emotionally-charged experiences). It is closer as well to how humans evolved, since during most of our history, we lived in small groups of kin people with reduced contacts with strangers. A second reason is that this approach allows more justified and fruitful comparisons with other species, and accordingly, enables to find more evolutionary continuity for human cognition and behavior (this discontinuity problem is discussed by e.g. Hackett 2017; Parravicini and Pievani 2018; Falzone 2019). Finally, since some basic processing strategies seem to be involved in all these types of linguistic diversity, the objective of achieving a truly unified neuroscience of linguistic variation appears as feasible. If succeeding in this, we will be in a favourable position to give a definitive answer to the conundrum posited by Evans and Levinson in their seminal paper (2009): why we are the only species with a communication system that is fundamentally variable at all levels.

That said, the kind of research we have advocated for here faces significant theoretical challenges, of the sort discussed in the paper, but also practical challenges of diverse nature. For instance, it is difficult to conduct experiments demanding neuroimaging facilities in most of the places where non-WEIRD languages are spoken. Likewise, tests and procedures might need some (extensive) adaptation. Recalling Ullman’s model of language processing, it happens that declarative memory is rather impacted by cultural factors. This is because it encompasses two subtypes of memory: episodic and semantic (Tulving 1972). Episodic memory allows to travel backwards and forwards in time, which is an universal ability of the human mind. By contrast, semantic memory stores word meanings, and what is lexicalized varies from one language to another, and ultimately from one culture to another. As a consequence, tests employed for measuring semantic memory abilities are expected to be culturally-biased to some degree and demand some adaptation (see Benítez-Burraco et al. 2024 for discussion). For instance, since these tests typically ask participants to name objects or animals displayed in a screen or a piece of sheet, one can choose objects or animals that are familiar to the participants, instead of using the original ones more suited to speakers of WEIRD languages. Even better, one can use images that are abstract and non-representational by nature (see Benítez-Burraco et al. 2024 for details).

A second aspect to be improved is the "logistics" of multidisciplinary research. It is crucial to think about more efficient ways of bringing together researchers from different fields, with diverse theoretical backgrounds and methodological skills, but also with their idiosyncratic limitations. For this, we can build on experiences by other fields of research. Language evolution could be used as a fruitful example. To understand how language evolved, linguists, anthropologists, archaeologists, ethologists, and many other scholars are increasingly collaborating in big research projects aimed at clarifying selected aspects of this big puzzle. A notable case of this fresh approach is the recent paper by Blasi et al. (2019), who provided an answer to the long-lasting enigma in historical linguistics of why Proto-Indo-European lacked labiodental sounds, despite being frequent in present-day Indo-European languages. The authors show that these sounds became frequent only when changes in the human bite anatomy made them easier to produce. In turn, such changes were triggered by cultural innovations: the transition from a meat-based diet to a grain-based diet after the adoption of agriculture resulted in an increased involvement of the molars in mastication, hence the changes in the bite systems.

Overall, if the theoretical and methodological issues discussed in the paper are properly addressed and fixed, we will certainly see in the next years notable advancements in our understanding of the neurobiological and cognitive basis of language diversity and the nature of language.

## Data Availability

The materials used for this research are presented as a list of bibliographical references at the end of the paper.

## Declarations

### Geolocation

Seville, Spain, Europe.
